# Supplementary material for: Existence of multiple critical cooling rates which generate different types of monolithic metallic glass
Source: Nat Commun. 2019 Mar 22;10:1337. doi: 10.1038/s41467-018-07930-3 (PMC6430809; doi:10.1038/s41467-018-07930-3)
Supplement: Supplementary file 1 — Supplementary Information [file 41467_2018_7930_MOESM1_ESM.pdf]

Supplementary Information

Existence of multiple critical cooling rates which generate different types of monolithic metallic glass

Jürgen E.K. Schawe[1] & Jörg F. Löffler[2]

Supplementary Note 1: Glass transition and crystallinity

Supplementary Figure 1 shows the relative intensity of the glass transition, $\Delta c_p/\Delta c_{p,\mathrm{a}}$, versus crystallinity α. It illustrates a linear correlation between the normalized glass transition intensity, $\Delta c_p/\Delta c_{p,\mathrm{a}}$, and the normalized total enthalpy of phase transformations, $\alpha = \Delta H/\Delta H_\mathrm{m}$, as expected for semi-crystalline material with a single type of amorphous phase.

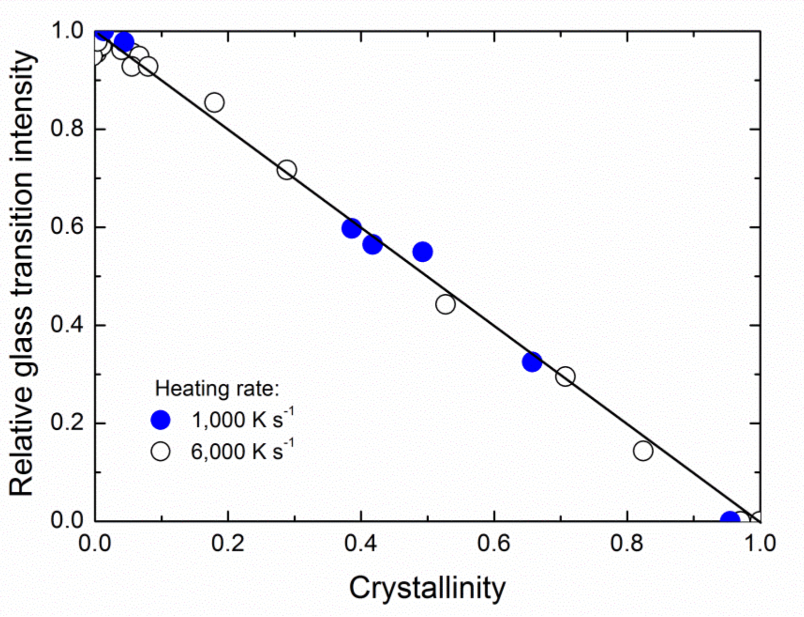

Supplementary Figure 1 | Relation between amorphous and crystalline phases. Relative intensity of the glass transition, $\Delta c_p/\Delta c_{p,\mathrm{a}}$, versus crystallinity $\alpha = \Delta H/\Delta H_\mathrm{m}$, which corresponds to the relative total enthalpy of the phase transitions.

Supplementary Note 2: SDGs and CHGs in a Pt-based glass

Supplementary Fig. 2 shows measurements on the BMG-forming alloy $Pt_{57.3}Cu_{14.6}Ni_{5.3}P_{22.8}$.

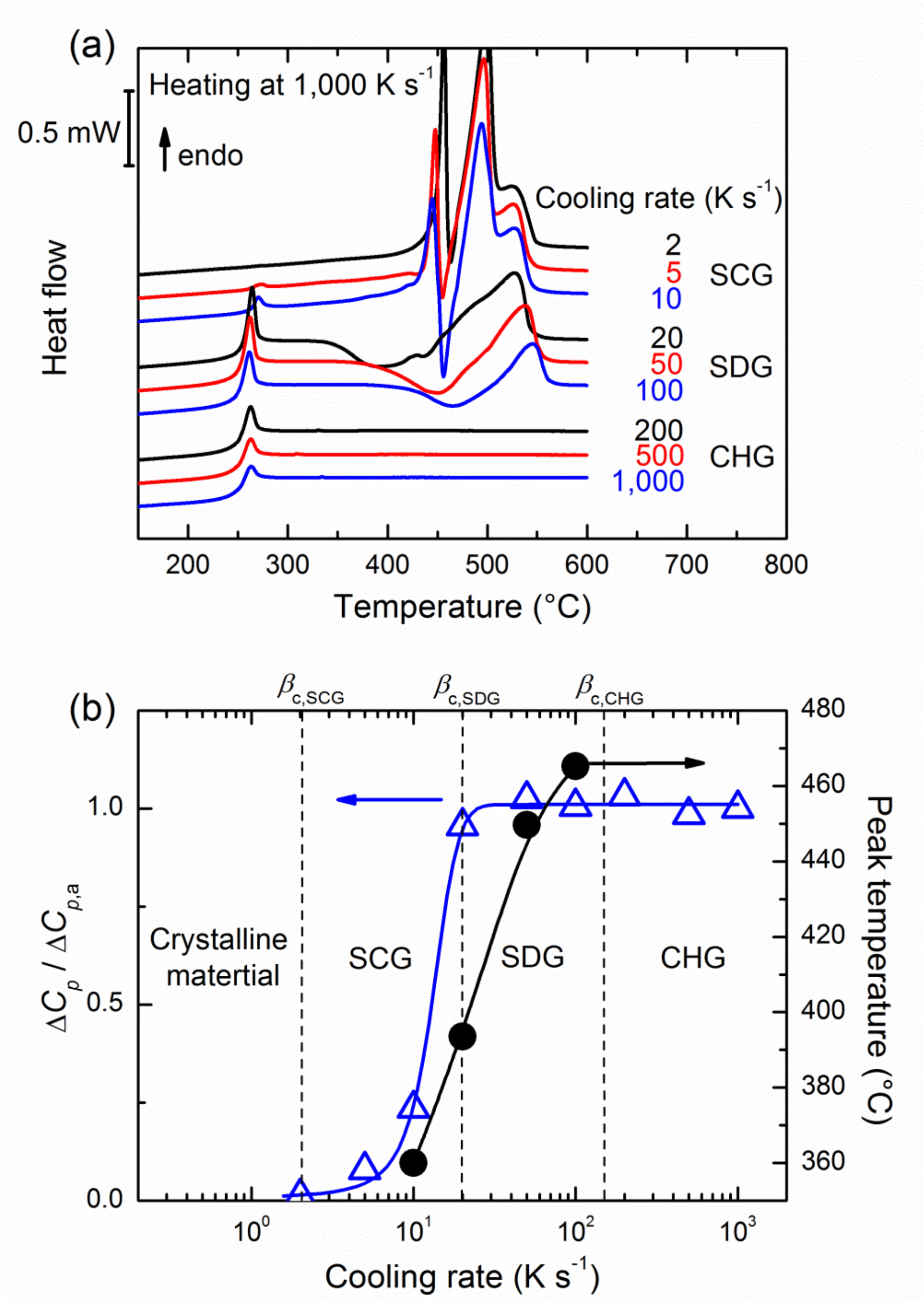

Supplementary Figure 2│ Thermophysical properties of Pt-based BMG. (a) Heating curves at 1,000 K s^{-1} after previous cooling at the indicated rates. The curves are grouped into CHG, where no crystallization or melting occurs (cooling rate: 200–1,000 K s^{-1}); SDG, where crystallization takes place before melting (20–100 K s^{-1}); and SCG, where the glass transition intensity decreases (2–10 K s^{-1}).
(b) Cooling rate dependence of the relative glass transition intensity (ΔC_p / $\Delta C_{p,\mathrm{a}}$) and the temperature of the first crystallization peak.

The Pd-based sample had a mass of approximately 0.8 μg and was cooled at rates between 2 and 1,000 K s^{-1} with subsequent heating at β_h = 1,000 K s^{-1}. The corresponding heating curves are shown in Supplementary Fig. 2a.

The glass transition and melting events were analyzed according to the procedure described in Figs. 1 and 2 in the main text. Supplementary Fig 2b shows the relative glass transition intensity, $\Delta C_p/\Delta C_{p,a}$, and the peak temperature of the crystallization peak as a function of the previous cooling rate. Completely crystalline material (without a glass transition), semi-crystalline glass (SCG), self-doped glass (SDG), and chemically homogeneous glass (CHG) can be identified and distinguished, in analogy to the Au-based alloy described in the main text. The critical cooling rates observed are $\beta_{c,SCG} \approx 2$ K s^{-1}, $\beta_{c,SDG} \approx 20$ K s^{-1}, and $\beta_{c,CHG} \approx$ 150 K s^{-1}, respectively. For CHG, up-quenching occurs because $\beta_h \geq \beta_{h,CHG}$.

Supplementary Note 3: Isothermal crystallization data

Supplementary Fig. 3 shows the heat-flow curves of the isothermal crystallization process after rapid heating from the glassy state. The cooling rate for forming the glass was selected such that a CHG (20,000 K s^{-1} in Supplementary Fig. 3a) or an SDG (500 K s^{-1} in Supplementary Fig. 3b) formed. The onset and peak times of crystallization fluctuate due to stochastic nucleation. To illustrate this we show two measuring curves for the isothermal hold at 294 °C. As expected, fluctuation for the CHG is greater than for the SDG.

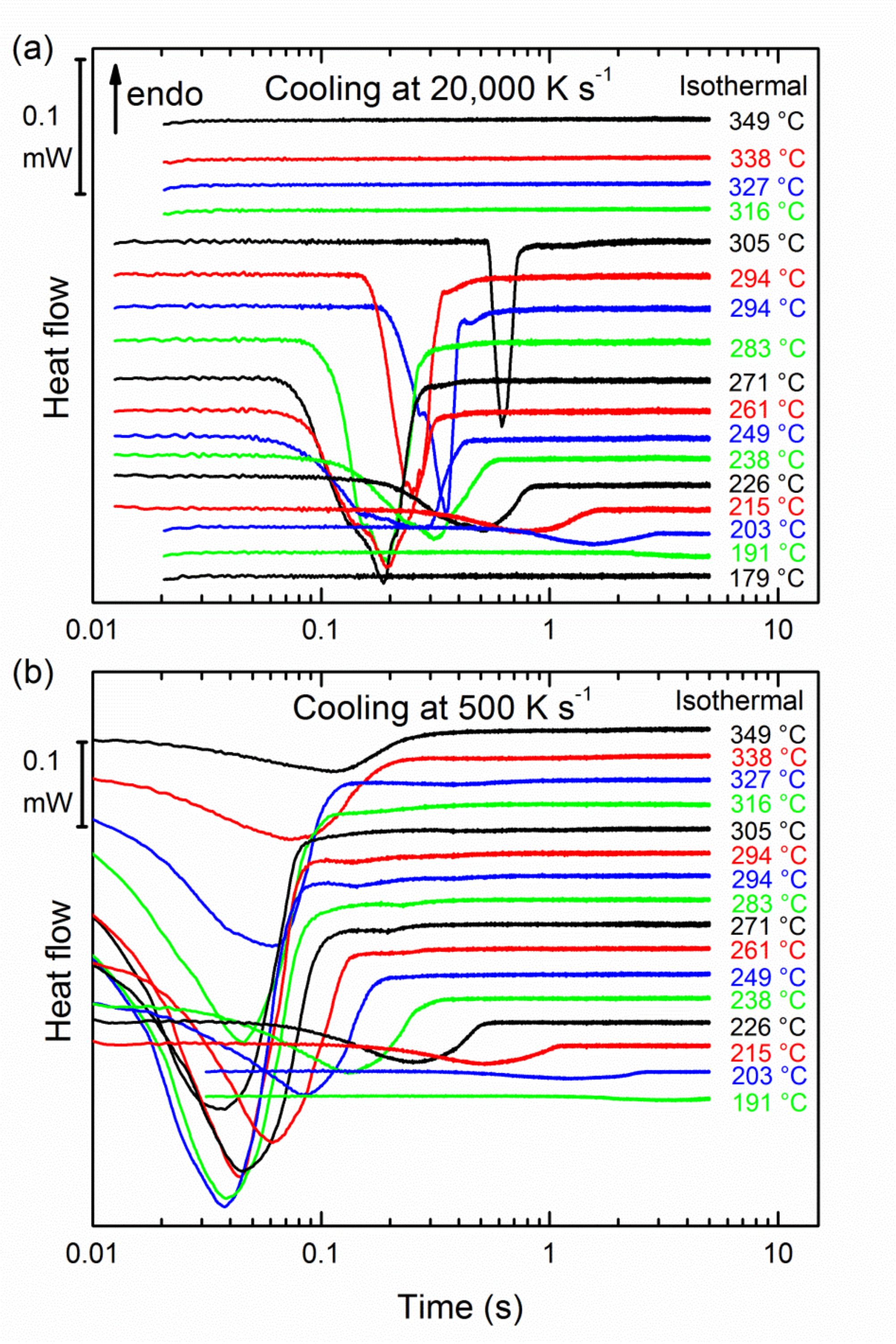

Supplementary Figure 3 | Isothermal crystallization. Isothermal crystallization curves for (a) CHG, obtained upon cooling to RT at a rate of 20,000 K s^{-1}, and (b) SDG, obtained by cooling to RT at a rate of 500 K s^{-1}. Heating to the isothermal temperature was performed at a rate of 30,000 K s^{-1}.

Supplementary Methods

1. Temperature calibration

The measured temperature is corrected by $T = T_{meas} - \Delta T - \tau\, \beta_h$, where T_{meas} is the measured onset temperature, β_h is the heating rate, and ΔT is the temperature difference between the extrapolated onset temperature (for $\beta_h \to 0$) and the onset temperature of melting measured by conventional DSC (amounting to 345 °C).

Indium was used as a standard reference in addition to the Au-based alloy, to improve the temperature calibration. The indium sample was placed at the reference side of the sensor and measured at different rates to determine the static temperature difference, ΔT (for $\beta_h \to 0$), between indium's actual melting temperature (156.6 °C) and the measured onset temperature of melting. Consequently, the temperature scale was corrected using the two standard temperatures of indium and the Au-based alloy.

2. Thermal lag

The thermal lag of the sensor–sample system, τ, is determined by measuring the onset of the melting peak during heating after cooling the melt at a rate of 1 K s^{-1} (Supplementary Fig. 4). The slope of the linear relation between the measured onset temperature of melting and the heating rate is $\tau = 0.404$ ms. The thermal lag depends on the effective heat-transfer behavior of sensor and sample and their thermal contact between each other. For thermal-lag correction the measurements for the Au-based alloy were used over the whole temperature range. The total temperature correction was performed using the temperature correction procedure of the Mettler-Toledo STARe evaluation software.

3. Enthalpy determination using a Flash DSC2+ device with UFH1 sensor

The detection limit of enthalpy determination for small crystallization events depends on the reproducibility of the measuring curve, the signal-to-noise ratio of the heat-flow measurement and the width of the thermal event. The characteristic values are determined using selected heating curves measured at 6,000 K s^{-1} after cooling at various rates (Fig. 4b of the main text). The curves and evaluations are shown in Supplementary Fig. 5. The noise can be characterized by the peak-to-peak distance of a single curve. It is on the order of ±1 μW

(Supplementary Fig. 5b). The reproducibility of the curves, given as the maximum deviation of the heat flow in the melt obtained for 10 measurements, is on the order of ±1.5 μW (Supplementary Fig. 5b).

To determine the sensitivity of the device, the crystallization peak of a curve with first indication of a crystallization event (curve 1 in Supplementary Fig. 5a) is analyzed; this curve was measured after cooling at 4,000 K s^{-1}. For a better peak evaluation a curve with no indication of crystallization (measured after cooling at 10,000 K s^{-1}) was used as a base line (curve 2). The difference curve is shown in Supplementary Fig. 5c, and the peak area is 110.5 nJ. The figure illustrates that a peak with half of the area can still be identified and evaluated. The sensitivity of enthalpy determination is thus below 50 nJ, which means that for a sample mass of approximately 1 μg the detection limit is below 50 mJ g^{-1}. Thus, the resolution limit for detecting crystallization is on the order of 0.1% for the Au-based alloy, which has a total transformation enthalpy of 40.4 J g^{-1}. In general, the resolution limit depends on sample size, heating rate and temperature range. The sample thickness and the thermal contact between sample and sensor may also influence the resolution and reproducibility of the measurements.

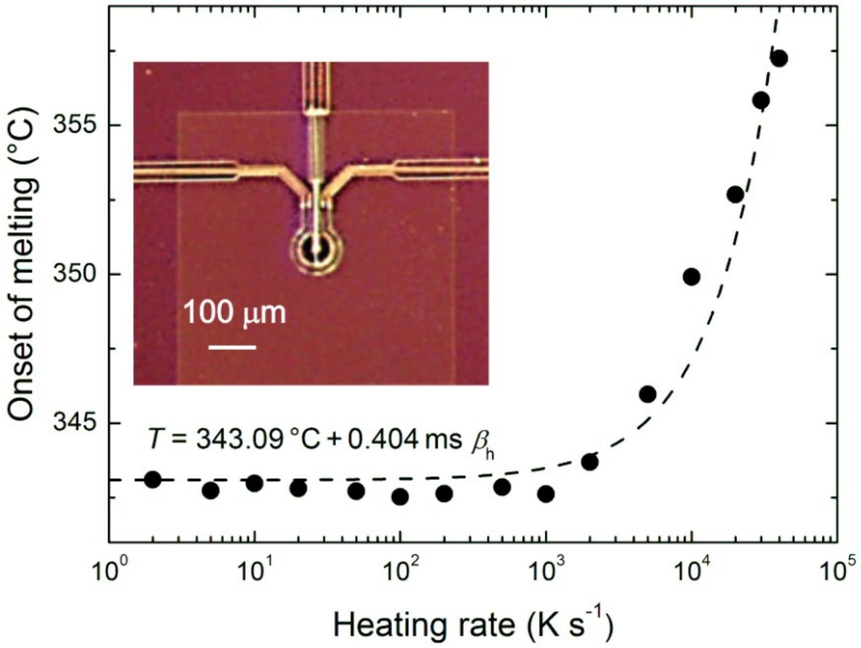

Supplementary Figure 4 | Thermal lag. Melting temperature (onset of melting peak) versus heating rate to determine the thermal lag of the sensor–sample system. The insert shows the sample side of the high-temperature sensor UFH 1.

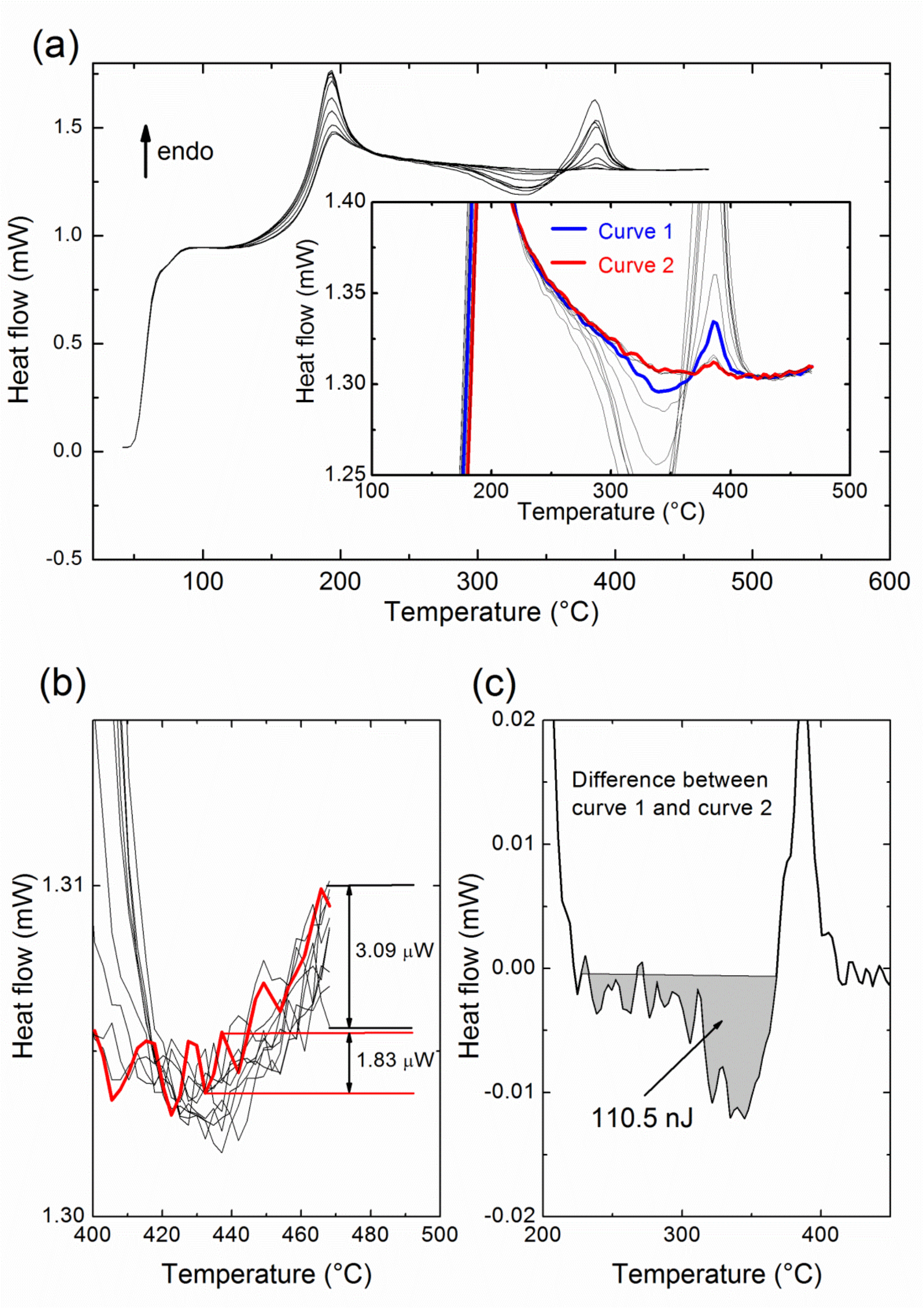

Supplementary Figure 5 │ Determining the resolution limit of the DSC2+ calorimeter. (a) Heating curves measured at 6,000 K s^{-1} after cooling at 1000–10,000 K s^{-1} (from Fig. 4b). The insert shows a magnified view of the crystallization and melting events; the curves after cooling at 3,000 K s^{-1} (curve 1) and 10,000 K s^{-1} (curve 2) are highlighted. (b) Indication of the noise and reproducibility of the measured curves. (c) Difference between curves 1 and 2 to evaluate the resolution limit for detecting crystallization.

Supplementary Discussion

1. Cooling rate dependence of peak temperature

To describe the cooling-rate dependence of the crystallization peak during heating, we use the Kolmogorov–Johnson–Mehl–Avrami (KJMA) equation for isothermal crystallization. In the case of heterogeneous crystallization it is

$$\alpha\ (t) = 1 - \exp\left(-g\, N R_{\mathrm{c}}^{n}\, t^{n}\right), \tag{S1}$$

where α is the crystallinity, g a geometry factor, N the nuclei number, R_{c} the growth rate, and n the Avrami exponent. In the simplest approach to crystallization during heating we substitute the growth rate by its average, $\langle R_{\mathrm{c}} \rangle$, and the time by $t = (T - T_1)\, \beta_{\mathrm{h}}^{-1}$ (T_1 is the minimum crystallization temperature during heating and β_{h} is the heating rate):

$$\alpha\ (T) = 1 - \exp\left(-g\, N \langle R_{\mathrm{c}} \rangle^{n}\, \beta_{\mathrm{h}}^{-n}\, (T - T_1)^{n}\right). \tag{S2}$$

The temperature of the crystallization peak during heating follows the form $\left.\frac{\mathrm{d}^2\alpha}{\mathrm{d}T^2}\right|_{T_{\mathrm{Peak}}} = 0$, which gives:

$$T_{\mathrm{Peak}} = T_1 + \frac{\beta_{\mathrm{h}}}{\langle R_{\mathrm{c}} \rangle} \left(\frac{n-1}{n}\right)^{\frac{1}{n}} N^{-\frac{1}{n}}. \tag{S3}$$

The number of nuclei depends on the previous cooling conditions. If $\langle R_{\mathrm{N}} \rangle$ is the average nucleation rate in the temperature range between the melting temperature, T_{m}, and the glass transition temperature, T_{g}, the number of nuclei after cooling at a rate β_{c} can be assumed to be

$$N = \frac{\langle R_{\mathrm{N}} \rangle (T_{\mathrm{m}} - T_{\mathrm{g}})}{|\beta_{\mathrm{c}} - \beta_0|}, \tag{S4}$$

where β_0 is the minimum cooling rate for crystallization during heating. By combining equations (S3) and (S4) one obtains an empirical expression for the dependence of the crystallization peak upon heating on nuclei formation during the previous cooling process at rate β_{c}:

$$T_{\mathrm{Peak}} = T_1 + C|\beta_{\mathrm{c}} - \beta_0|^{\kappa}, \tag{S5}$$

where C and κ are empirical constants.
